# Factors affecting establishment and population growth of the invasive weed *Ambrosia artemisiifolia*

**DOI:** 10.3389/fpls.2023.1251441

**Published:** 2023-09-22

**Authors:** Wenxuan Zhao, Zhifang Xue, Tong Liu, Hanyue Wang, Zhiquan Han

**Affiliations:** ^1^ College of Life Science, Shihezi University, Shihezi, China; ^2^ Xinjiang Production and Construction Corps Key Laboratory of Oasis Town and Mountain-basin System Ecology, Shihezi, China; ^3^ College of Science, Shihezi University, Shihezi, China

**Keywords:** propagule pressure, Allee effect, aggregation effect, invasion probability, invasion threshold

## Abstract

*Ambrosia artemisiifolia* is a highly invasive weed. Identifying the characteristics and the factors influencing its establishment and population growth may help to identify high invasion risk areas and facilitate monitoring and prevention efforts. Six typical habitats: river banks, forests, road margins, farmlands, grasslands, and wastelands, were selected from the main distribution areas of *A. artemisiifolia* in the Yili Valley, China. Six propagule quantities of *A. artemisiifolia* at 1, 5, 10, 20, 50, and 100 seeds m^-2^ were seeded by aggregation, and dispersion in an area without *A. artemisiifolia*. Using establishment probability models and Allee effect models, we determined the minimum number of seeds and plants required for the establishment and population growth of *A. artemisiifolia*, respectively. We also assessed the moisture threshold requirements for establishment and survival, and the influence of native species. The influence of propagule pressure on the establishment of *A. artemisiifolia* was significant. The minimum number of seeds required varied across habitats, with the lowest being 60 seeds m^-2^ for road margins and the highest being 398 seeds for forests. The minimum number of plants required for population growth in each habitat was 5 and the largest number was 43 in pasture. The aggregation distribution of *A. artemisiifolia* resulted in a higher establishment and survival rate. The minimum soil volumetric water content required for establishment was significantly higher than that required for survival. The presence of native dominant species significantly reduced the establishment and survival rate of *A. artemisiifolia*. *A. artemisiifolia* has significant habitat selectivity and is more likely to establish successfully in a habitat with aggregated seeding with sufficient water and few native species. Establishment requires many seeds but is less affected by the Allee effect after successful establishment, and only a few plants are needed to ensure reproductive success and population growth in the following year. Monitoring should be increased in high invasion risk habitats.

## Introduction

The successful establishment and reproduction of invasive alien plants after entering new habitats are essential for the continuation and rapid growth of the population (Warren and Bradford, 2011). Clarifying the regularity, characteristics, and influencing factors of the establishment and population growth of invasive plants and estimating the invasive risk in various habitats can facilitate the identification of high invasion risk areas, leading to timely monitoring, early warning, prevention, and control treatment (Gertzen et al., 2011; Zhao et al., 2020).

Invasive alien plants that spread to new habitats require sufficient seed numbers to establish and grow and survive (Colautti et al., 2006). Sexually reproducing species also need to overcome the lack of mates and effective pollinators in small populations in order to reproduce successfully (Courchamp et al., 2008). At the same time, native species and abiotic environmental factors (moisture, temperature, etc.) have a continuous impact on establishment, reproduction, and population growth (Visscher et al., 2023).

The propagule pressure hypothesis suggests that the number of propagules introduced into a new habitat and the frequency of introduction events are among the most important factors influencing the success of plant invasion (Simberloff, 2009). The number of propagators entering a new habitat directly affects the probability of survival and determines the success of establishment of invasive alien plants (Lockwood et al., 2009; Williams et al., 2021). Meanwhile, since resources are fully available during the very early phases of colonization, most propagule numbers can rapidly preempt or modify the available niche (You et al., 2016). Therefore, for successful establishment, the number of arriving individuals must be above an ‘invasion threshold’ (Lewis and Kareiva, 1993) defined as the minimum number required for establishment to occur.

After establishment, as the population is currently small, the influence of the Allee effect is more prominent (Allee, 1931). In sexually reproducing invasive alien plants, the Allee effect is primarily driven by the fewer potential mates and effective pollinators (Courchamp et al., 2008). Determining whether the Allee effect exists in an established population and the minimum number of plants that can achieve successful reproduction and facilitate population growth can provide a theoretical basis for the scientific development of efficient and low-cost prevention and control strategies (Berec et al., 2007; Tobin et al., 2011).

Propagules that spread to new habitats must also overcome environmental constraints, such as water availability and temperature. Additionally, competition from a resident community may reduce invasion capacity (Levine et al., 2004). Although there is little evidence suggesting that species interactions can completely resist invaders, some resident communities can reduce the establishment, abundance, and fitness of invaders to a certain extent (Liu et al., 2014). Therefore, identifying the effects of environmental factors on the establishment and population growth of invasive alien plants can provide an important basis for determining the invasiveness of habitats.

Predicting the establishment of exotic species is a central aim of invasion biology and depends on propagule pressure and population processes (Rojas-Botero et al., 2022). Although the relationship between the number of invasive plant propagules and invasion success has been well investigated (Warren et al., 2012; Vedder et al., 2021), data on the minimum number of propagules required for the successful establishment of invasive alien plant populations are limited. Therefore, it is very important to determine the minimum number of seeds required for successful establishment and the minimum number of plants for population growth of invasive alien plants under the influence of different environmental factors to judge their invasion risk.

It is useful to summarize the rules of invasive plant establishment and population growth to accurately predict the risk of habitat invasion by determining the minimum number of propagules required for successful establishment and the minimum number of plants required for population growth (Catford et al., 2019). In areas of invaded natural habitats where the invader is not present, the simulated invasion of artificially sown propagules could more comprehensively identify the combined influence of multiple biological and abiotic factors on establishment success (Warren et al., 2012).

The invasive annual herbaceous species, *Ambrosia artemisiifolia*, is a troublesome plant in many regions, including Central and Eastern Europe and China (Essl et al., 2015; Montagnani et al., 2017). *A. artemisiifolia* produces a large amount of pollen, costing European healthcare systems approximately 7.4 billion euros in the treatment of associated allergies every year (Schafner et al., 2020). *A. artemisiifolia* can forms a persistent soil seed bank as a result of complex germination strategies (Essl et al., 2015). The influence of propagule pressure and disturbances on the distribution of *A. artemisiifolia* is greater than that of environmental factors (Dullinger et al., 2009; Urbanowicz et al., 2018). Kröel-Dulay et al. (2019) found that the invasion probability of *A. artemisiifolia* increases with increasing propagule pressure in continuously disturbed habitats. However, the minimum number of seeds required for successful establishment (MNS) and the minimum number of plants required for the population growth (MNP) of *A. artemisiifolia* have not been studied.

*A. artemisiifolia* relies on seeds to propagate, most of which spread around the plant at a close distance due to the action of both wind and gravity, while a small number of seeds spread over long distances through animal feeding or attachment to the surface of animal fur, along with rivers, or mixed in cargo (Essl et al., 2015). Seed dispersal models indicate that seed aggregation or dispersal may occur when reaching new habitats via long-distance dispersal (Cousens et al., 2008).

Therefore, we asked if there are differences in the establishment of the seeds of *A. artemisiifolia* when they are aggregated or dispersed after reaching new habitats and if and how established plants successfully reproduce for population continuity and growth. What factors influence the establishment and growth of *A. artemisiifolia*? We sowed *A. artemisiifolia* in six typical invasived habitats, namely, river banks, forests, road margins, farmland, grassland, and wasteland, invaded by *A. artemisiifolia* were selected in the Yili Valley, China. Areas without *A. artemisiifolia* distribution were artificially seeded to simulate long-distance dispersal of *A. artemisiifolia*.

In this study, we aimed to (1) determine the characteristics of propagule pressure and the MNS of *A. artemisiifolia* in each habitat; (2) Allee effect and fruiting characteristics of *A. artemisiifolia* to determine the MNP needed to achieve population growth; and (3) the effects of environmental factors (native species, temperature, and moisture) on establishment and population growth of *A. artemisiifolia*. This study determined the degree of difficulty in the establishment and population growth of *A. artemisiifolia* in the main invasive habitats. Our results provide an important theoretical basis for early monitoring and effective prevention and control measures to mitigate the damage associated with the invasion of *A. artemisiifolia*.

## Materials and methods

### Study area

The Yili Valley (42°14′–44°53′N, 80°09′–84°56′E) lies in the westernmost part of the Tianshan Mountain Range of Xinjiang. The Valley comprises 56,400 km^2^, and has a continental temperate arid climate. The region has an average annual temperature of 10.4°C and precipitation of 417.6 mm. In grassland habitats, which represent the wettest area in the Yili River, precipitation can reach 500 mm annually. The Yili Valley, with its rich plant diversity and extensive seed dispersal via canals, cattle, sheep, and tourists, provides favorable conditions for the invasion and rapid spread of invasive alien species (Jia et al., 2011).

### Experimental design

#### Seeds

The seeds used in this experiment were collected from complete plants in 8 A*. artemisiifolia* populations in different habitats in the invaded area of Yili Valley in September 2020 and the first batch of mature seeds were collected. Before the experiment, all immature, shriveled, worm-fed, and moldy seeds were removed; the remaining healthy mature seeds were thoroughly mixed, and several groups of 1, 5, 10, 20, 50, and 100 seeds were randomly selected for packaging. Therefore, the seeds used in the experiment were not subject to variations in germination rate and seed quality which could influence the results.

#### Plots setting

According to our previous investigation (Dong et al., 2020), there are six typical habitat types invaded by *A. artemisiifolia* in the Yili Valley: river banks, forests, road margins, farmland, grassland, and wasteland. There is virtually no *A. artemisiifolia* observed in other habitats. For this study the following habitats were used: the forest consisting of a shelterbelt on both sides of an agricultural road, the grassland on a hilly pasture, the wasteland was an abandoned farmland area (abandoned for five years), the farmland was artificial irrigated land, and the road margins were on both sides of the national road with heavy truck traffic.

In November 2020, an area with flat terrain, similar soil conditions and no intrusion and distribution of *A. artemisiifolia*, was selected as the experimental plot for each habitat. A 2 m high gauze was used to surround the experimental plot 2 m away from the experimental plot to avoid animal and human interference, and at the same time, the diffusion and invasion of *A. artemisiifolia* would not occur in the surrounding area of the experimental plot.

**Figure 1:**
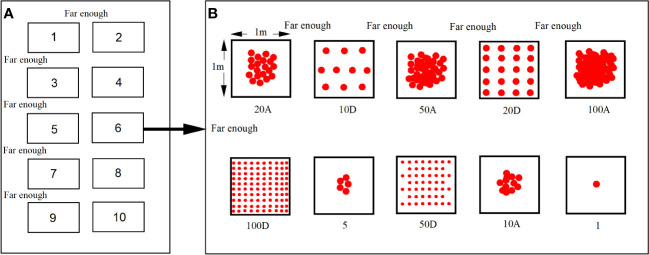
Schematic diagram of the experimental setting of propagule pressure with different seeding number and methods. **(A)** Ten replicates in every plot. **(B)** Seeding number and method of *Ambrosia artemisiifolia* in one repeated treatment. A: Aggregated seeding, D: Dispersed seeding.

The entire plot was divided into 10 cells sufficiently far apart as replicates for each treatment (**Figure 1A**). In each cell, 10 quadrats (1 m × 1 m) were set up as pressure treatments for the propagules (**Figure 1B**). To ensure that the same treatments were not adjacent, quadrats were randomly selected. The space between the quadrats in each treatment was sufficiently large to ensure no interaction between quadrats.

In the central area of each plot, a soil temperature and humidity recorder (WatchDog 1400 Data Logger) was installed at a position with stable terrain and no fluctuation. Temperature and humidity changes were recorded continuously at hourly intervals every day as habitat temperature and humidity data, respectively. After the experiments, the data were returned to the laboratory for analysis.

### Artificial sowing quantity and sowing method setting

The introduction of invasive plant propagules is a random event; therefore, the number of propagules introduced into new habitats is usually not large. By observing the newly diffused ragweed population in the Yili Valley and referring to previous studies (Deng et al., 2016; Kröel-Dulay et al., 2019), we identified six reproductive number treatments: 1, 5, 10, 20, 50, and 100 seeds m^-2^.

Considering the aggregation and dispersal of *A. artemisiifolia* seeds that have spread into new habitats (Houseman, 2014), two sowing methods were adopted: (1) Aggregation: All seeds were concentrated in the central area of the quadrat to simulate a relatively concentrated distribution of seeds post mixing in goods or being swallowed by animals, collected in feces, discharged from the body, and dispersed into new habitats. (2) Dispersion: All seeds were evenly spread throughout the quadrat to simulate the relatively dispersed distribution of seeds after they were attached to animal fur or dispersed into new habitats by vehicles, rivers, or other media. Since the numbers of seeds treated with one and five were too small, a dispersed seeding treatment was not set up.

A total of 600 quadrats were used in the experiment: 6 habitats× [(6 × 10 propagule treatments) + (4 × 10 seed treatments)].

### Measurements

Due to continuous germination, for the maximum number of established plants in each treatment, a large number of seedling stage plants were concentrated near the end (SS; on April 25 ± 3 days, 2021). The number of *A. artemisiifolia* plants in the quadrat was investigated, and the establishment rate under each treatment was calculated (the number of established plants/the number of seeds sown in the treatment).During the mature stage of *A. artemisiifolia* (MS; September 15 ± 3 days, 2021), the number of surviving plants in the quadrat was counted and the survival rate under each treatment was calculated (the number of surviving plants in the setting stage/the number of established plants in the SS). Furthermore, the number of seeds per plant of all surviving *A. artemisiifolia* in each treatment was counted, and the total number of seeds in the population was calculated (sum of seeds per plant of all *A. artemisiifolia* in the quadrat). If some plants lost seeds, the number of pistil clusters and the location of the seeds were estimated.

To clarify the effects of native species on the establishment and survival of *A. artemisiifolia*, the number (total number of individuals in the quadrat for general plants and tillering number for gramineous plants), frequency, coverage, and plant height of *A. artemisiifolia* and the native species in each quadrat were counted during these two periods. The relative abundance (RA), relative coverage (RC), relative frequency (RF), relative height (RH) of *A. artemisiifolia* and native species were calculated for each habitat during SS and MS (**Supplementary Table A.1**).

To determine the growth capacity of the *A. artemisiifolia* population, the newly established *A. artemisiifolia* in each treatment quadrat was investigated at the SS of the following year (April 25 ± 3 days, 2022).

At the end of all experiments, all *A. artemisiifolia* in the plots were subjected to continuous chemical treatment (Wang et al., 2022), to ensure their removal. The surrounding areas were monitored and maintained for two consecutive years to ensure that no *A. artemisiifolia* had spread into the surrounding habitat and caused new invasions.

### Model

#### Probability model of successful establishment of *A. artemisiifolia*

The density-dependent invasion pressure model constructed by Barney et al. (2016) is as follows:


(1) $$Y=1-{\left(1-PN^{D}\right)}^{N}$$

where *Y* is defined as the invasion pressure or the probability that at least one propagule is established in a given area, *P* is the establishment probability of a single propagule in a given area, *N* is the number of propagules arriving in a given area in an introduction event, and *D* is a density dependence parameter that is expected to fall between 0 and -1.

In the present study, under different seeding treatments, the seeds of *A. artemisiifolia* were affected by the number and seeding method (aggregation/dispersion), and the density-dependent effect was different, which further affected the final establishment probability. Therefore, we used Equation (1) to consider the effect of density dependence on the establishment probability of *A. artemisiifolia* with different propagule numbers.

Through experimental observation, the establishmet rate *P* of *A. artemisiifolia* in different habitats under different treatments was obtained, and the slope *D* of the survival curve of *A. artemisiifolia* population in the SS under different *N* treatments (1, 5, 10, 20, 50, and 100 seeds m^-2^) was calculated. Then, 100 groups of data observed in each habitat in the experiment (sowing number 6× repeat 10+ sowing method 4× repeat 10) were substituted in Equation (1) to calculate the *Y* values under different treatments and solve each parameter to determine the relationship between the invasion and establishment probability of *A. artemisiifolia* and the number of propagators. The *N* value at *Y* = 1 was determined by fitting; therefore, the MNS of *A. artemisiifolia* invading and established in each habitat of Yili Valley was obtained.

### Allee effect model of *A. artemisiifolia* population

We referred to Tobin et al. (2011) to consider the effects of different treatments (removal and application of pesticides, among others) on the number of invasive populations and constructed the following model:


(2) $$N_{t+1}=\lambda \left(1-P\right)N_{t}\frac{\left(1-P\right)N_{t}}{\left(1-P\right)N_{t}+\theta }$$

where the population density *N_t+1_* at time t+1 equals the per-capita fecundity *λ* multiplied by the population density *N_t_* at time t times the fraction *N_t_*/(*N_t_* + *θ*) of individuals that succeed. In identifying mates and reproduce; *θ* is the Allee effect strength. It is assumed that a fraction *P* (0< *P*< 1) of individuals dies.

In this study, due to various biological and abiotic factors, the *P* ratio of the individual deaths of *A. artemisiifolia* surviving after successful establishment under different propagation pressures was also observed; this effect was continuous with the growth of *A. artemisiifolia*. Here, we redefined *P* as the proportion of *A. artemisiifolia* dying from establishment to fruiting but retained its expression.

Therefore, for *A. artemisiifolia* which survives and bears fruit after establishment, population density in the second year can be expressed using Equation (2). The Allee effect threshold for *A. artemisiifolia* population was


(3) $$A_{p}=\frac{\theta }{\left(1-P\right)\left[\left(1-P\right)\lambda -1\right]}$$

In other words, after successful establishment, the population density of A. artemisiifolia could not achieve effective population growth when it was lower than *A_p_*, and the population growth rate *N_t_*_+1_/*N_t_* was less than 1–0.

In this study, the number of surviving *A. artemisiifolia* plants (1-*P*)*N_t_*, the number of seed *λ*, and the number of newly settled *A. artemisiifolia* plants *N_t_*_+1_ at the second SS of invasion under different treatments in each habitat were experimentally observed. A total of 100 groups of data (sowing number 6× 10 repetitions + sowing method 4× 10 repetitions) observed in each habitat in the experiment were substituted into Equations (2) and (3) to calculate the *θ* value and threshold *A_p_* of the Allee effect under different treatments and solve for the parameters to determine the relationship between population growth and plant number after the establishment of *A. artemisiifolia*. Then, the Allee effect threshold *A_p_* is calculated. By fitting the *N_t_* value at an *N_t_*_+1_/*N_t_* value of 1, the MNP required for the successful reproduction and population growth of *A. artemisiifolia* in each habitat in the Yili Valley was obtained.

### Establishment and survival with water and temperature requirements of *A. artemisiifolia*

For the soil volume, water content, and temperature data obtained from each habitat, the temperature and humidity data for the initial 30 days of observation were selected for the growth period. Furthermore, the average value of the temperature and humidity values recorded every day (once every hour, 12 groups in total) were considered as the temperature and humidity of the day, for a total of 30 treatments, with an average value of the temperature and humidity of the growth period.

The coefficient of variation (CV) was calculated to reflect the degree of fluctuation in soil temperature and humidity during different growth periods (**Supplementary Table A.2**).

To clarify the effects of soil volumetric water content and temperature on the settlement and survival of *A. artemisiifolia* and determine the threshold range and optimal value of soil volumetric water content and temperature required for the settlement and survival of *A. artemisiifolia*, A Gaussian regression model was used to fit the soil volumetric water content and temperature with the establishment and survival rates of *A. artemisiifolia* at SS and MS, respectively.

The establishment and survival rates were fitted to soil volumetric water content or temperature, respectively, using a unitary quadratic function. If the unitary quadratic function is in line with these two relationships, a Gaussian relationship can be determined (Zhang, 2004). The quadratic equation of a variable with a Gaussian relation is converted into a Gaussian regression equation as follows:


(4) $$Y=A\text{ exp }\left[-0.5{\left(x-\mu \right)}^{2}/t^{2}\right]\;$$

where *Y* is the establishment or survival rate of all the seeding treatments in the plot, *A* is the maximum establishment or survival rate of *A. artemisiifolia*, *μ* is the soil volume water content or temperature value when the establishment rate or survival rate of *A. artemisiifolia* peaks, that is, the optimal value of environmental factors, and *t* is the tolerance of *A. artemisiifolia*, the higher the *t* value, the wider the range of *A. artemisiifolia* adaptation to this environmental factor. The ecological threshold interval of soil volume water content and temperature of *A. artemisiifolia* was[*μ*−2*t*, *μ*+2*t*], and the optimal ecological threshold interval was [*μ*−*t*, *μ*+*t*]. Therefore, the key to determining the threshold of soil volume water content and temperature for the *A. artemisiifolia* establishment and survival is to determine the values of *μ* and *t*.

The species distribution data of the Global Biodiversity Information Facility (GBIF) were searched using the R software to identify the water and temperature characteristics and variation in *A. artemisiifolia* in North America, Europe, and the Yili Valley, in native and invaded ranges.

Erroneous or duplicate records and filter data were removed, and the latitude and longitude information of the species distribution points were used to extract climate variables from the WorldClim database for analysis and mapping (**Supplementary Figure A.1**).

### Statistical analysis

We used the SPSS software, data were tested for normality using the “QQ plot” in the “descriptive statistics”, and then distributed in a straight line for each treatment. All data followed a normal distribution. Further, data were tested for homogeneity. If the variance difference of each group was not significant (*p* > 0.05), the data conformed to the homogeneity of variance.

We used generalized linear mixed models (GLMM) and selected normal distribution model constant function to evaluate the fixed effects (seeding number, seeding method, and seeding number × seeding method) on establishment rate, survival rate and total seed number. We accounted for the influence of native species, by evaluating sum of the RA, RC and RH of the native species and the interaction between them (RA × RC, RA × RH, RC × RH, RA × RC × RH) as random effects in the GLMMs. The mixed models were fit using the Laplace approximation in the ‘lme4’ package (Bates and Maechler, 2009) of the R statistical programming environment (R.V4.1.1). The inclusion or exclusion of the random effects was based on Akaike’s information criterion (AIC) values (Akaike, 1973). AIC measures the distance of models to truth; that is, the information loss in the data given that the model is a simplification of reality and cannot explain full variance in the data (Burnham and Anderson, 2002). In effect, AIC is a measure of goodness-of-fit, as the more familiar R^2^ measure, with an added penalty for model complexity as measured by the number of fit parameters in the model.

To examine the impact of native species traits on the establishment rate and survival rate of *A. artemisiifolia*, Pearson correlation analyses of “sum of the RA of the native species”, “sum of the RC of the native species” and “sum of the RH of the native species” and “establishment/survival rate” data were conducted for each habitat. Therefore, some factors showed a significant correlation, while others showed no significant correlation.

ANOVA with Duncan’s method was used to compare the differences in establishment and survival rate of *A. artemisiifolia* under different seeding number and seeding methods, as well as the differences in the total number of seeds and the number of new plants in the next year.

## Results

### Factors affecting establishment, survival, and total seed number

**Figure 2:**
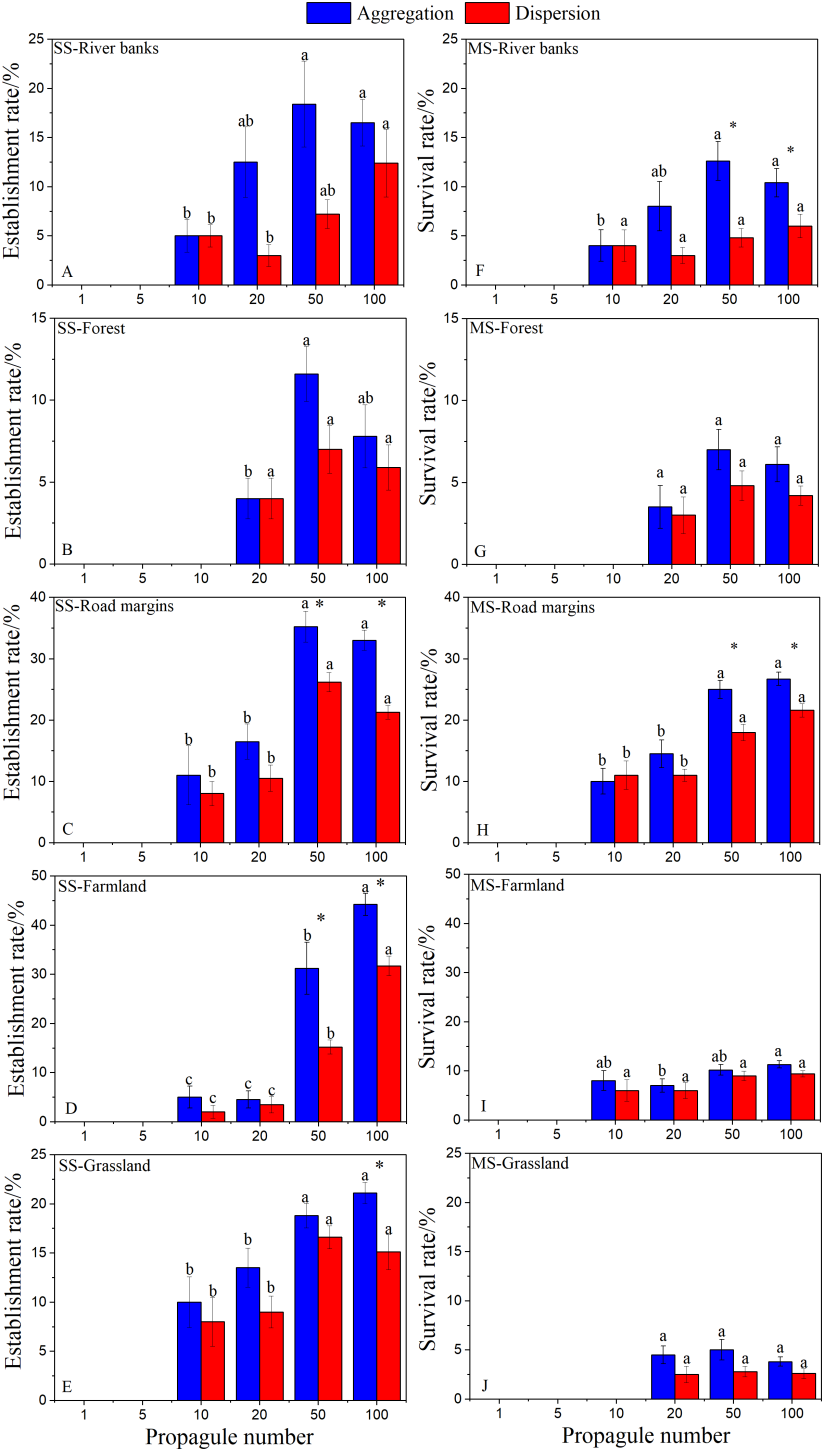
Effects of sowing number and method on establishment rate **(A–E)** and survival rate **(F–J)** of *Ambrosia artemisiifolia* in each habitat (Mean ± SE). Different letters indicate significant difference in seeding number under the same seeding method (*P*< 0.05), * indicated that there were significant differences among different seeding methods under the same seeding number (*P*< 0.05). Since there was no *A. artemisiifolia* germination in the wasteland, no data was obtained, so it was not shown in the figure.

The GLMM test results (**Table 1**) showed that the seeding number, seeding method, and their interaction had significant effects on the establishment and survival rates of *A. artemisiifolia* in each habitat (**Figure 2**). The total number of seeds in the population was mainly affected by the seeding number and seeding method (**Tables 2**, **3**).

**Table 1:** Test results of GLMM on the effects of various factors on establishment rate, survival rate and total seed number of population of *Ambrosia artemisiifolia*.

Habitats	Factors	Establishment rate			Survival rate			Total seed number		
		*Wald*	*df*	*P*	*Wald*	*df*	*P*	*Wald*	*df*	*P*
River banks	Fixed effect									
	1. Seeding number	3.426	3	**0.033**	12.018	3	**0.007**	21.679	3	**<0.001**
	2. Seeding method	9.036	1	**<0.001**	5.640	1	**0.018**	9.472	1	**0.002**
	3. Number × Method	17.866	3	**<0.001**	18.685	3	**<0.001**	5.305	3	0.151
	Random effect									
	1. RA	5.234	1	**0.022**	6.420	1	**0.011**	0.546	1	0.979
	2. RC	0.960	1	0.327	0.569	1	0.291	0.518	1	0.985
	3. RH	1.042	1	0.307	0.541	1	0.318	0.479	1	0.990
	4. RA × RC	0.839	1	0.360	0.489	1	0.357	0.503	1	0.984
	5. RA × RH	0.899	1	0.343	0.433	1	0.410	0.431	1	0.996
	6. RC × RH	0.986	1	0.321	0.525	1	0.332	0.480	1	0.990
	7. RA × RC× RH	0.856	1	0.355	0.331	1	0.451	0.541	1	0.976
Forest	Fixed effect									
	1. Seeding number	20.189	3	**<0.001**	9.767	3	**0.008**	14.782	3	**0.001**
	2. Seeding method	10.101	1	**<0.001**	7.493	1	**0.024**	10.222	1	**0.001**
	3. Number × Method	6.688	3	**0.043**	5.872	3	**0.032**	3.366	3	0.186
	Random effect									
	1. RA	5.999	1	**0.014**	6.223	1	**0.013**	0.822	1	0.365
	2. RC	5.564	1	**0.018**	5.900	1	**0.015**	0.896	1	0.344
	3. RH	0.313	1	0.576	0.634	1	0.426	0.963	1	0.327
	4. RA × RC	4.985	1	**0.026**	6.372	1	**0.012**	0.863	1	0.353
	5. RA × RH	0.326	1	0.568	0.607	1	0.436	0.926	1	0.336
	6. RC × RH	0.295	1	0.587	0.662	1	0.416	1.007	1	0.316
	7. RA × RC× RH	0.306	1	0.580	0.633	1	0.426	0.971	1	0.324
Road margins	Fixed effect									
	1. Seeding number	32.354	3	**<0.001**	24.376	3	**<0.001**	463.52	3	**<0.001**
	2. Seeding method	13.231	1	**<0.001**	18.937	1	**<0.001**	22.447	1	**<0.001**
	3. Number × Method	9.864	3	**0.020**	10.140	3	**<0.001**	37.552	3	**<0.001**
	Random effect									
	1. RA	5.352	1	**0.029**	6.872	1	**0.022**	1.003	1	0.317
	2. RC	1.006	1	0.316	0.270	1	0.603	1.002	1	0.317
	3. RH	0.998	1	0.318	0.259	1	0.611	1.060	1	0.303
	4. RA × RC	0.853	1	0.356	0.301	1	0.583	0.984	1	0.321
	5. RA × RH	0.846	1	0.358	0.289	1	0.591	1.042	1	0.307
	6. RC × RH	1.046	1	0.306	0.276	1	0.600	1.041	1	0.308
	7. RA × RC× RH	0.888	1	0.346	0.307	1	0.579	1.023	1	0.312
Farmland	Fixed effect									
	1. Seeding number	18.380	3	**<0.001**	48.652	3	**<0.001**	196.63	3	**<0.001**
	2. Seeding method	19.019	1	**<0.001**	6.414	1	**0.011**	2.211	1	0.137
	3. Number × Method	28.220	3	**<0.001**	6.945	3	**0.027**	2.336	3	0.506
	Random effect									
	1. RA	4.197	1	**0.040**	3.604	1	**0.036**	0.421	1	0.516
	2. RC	0.871	1	0.792	3.413	1	0.058	0.366	1	0.545
	3. RH	0.489	1	0.520	3.566	1	0.059	0.303	1	0.582
	4. RA × RC	0.452	1	0.438	3.495	1	0.062	0.433	1	0.511
	5. RA × RH	0.377	1	0.588	3.456	1	0.063	0.360	1	0.548
	6. RC × RH	0.523	1	0.316	3.640	1	0.056	0.312	1	0.576
	7. RA × RC× RH	0.269	1	0.210	3.529	1	0.060	0.371	1	0.542
Grassland	Fixed effect									
	1. Seeding number	26.520	3	**<0.001**	60.848	3	**<0.001**	28.390	3	**<0.001**
	2. Seeding method	18.687	1	**<0.001**	19.274	1	**<0.001**	6.918	1	**0.009**
	3. Number × Method	8.459	3	**0.037**	8.44	3	**0.015**	1.756	3	0.416
	Random effect									
	1. RA	5.666	1	**0.027**	5.136	1	**0.017**	0.157	1	0.691
	2. RC	0.358	1	0.550	0.789	1	0.374	0.173	1	0.677
	3. RH	0.419	1	0.518	4.892	1	**0.033**	0.195	1	0.659
	4. RA × RC	0.409	1	0.523	0.773	1	0.379	0.160	1	0.689
	5. RA × RH	0.474	1	0.491	3.318	1	**0.039**	0.182	1	0.670
	6. RC × RH	0.291	1	0.589	0.803	1	0.370	0.198	1	0.657
	7. RA × RC× RH	0.336	1	0.562	0.787	1	0.375	0.184	1	0.668

RA: Total relative abundance of native species. RC: Total relative coverage of native species. RH: Total relative plant height of native species. Bold indicates significant effect.

**Table 2:** Total seed number of *Ambrosia artemisiifolia* population in different habitats under different treatments (seeds m^-2^, Mean ± SE).

Habitats	Seeding number	Seeding method		Habitats	Seeding number	Seeding method	
		Aggregation	Dispersion			Aggregation	Dispersion
River banks	1	/	/	Farmland	1	/	/
	5	/	/		5	/	/
	10	23.25±4.15c	26±3.58b		10	42.43±8.71c	30.2±2.76c
	20	93.71±16.35c	44.33±6.54b		20	45.67±7.28c	46.68±7.59c
	50	561.1±109.69b	138.8±33.15b		50	338.3±44.23b	277±32.4b
	100	1073.8±210.53a	430.22±82.08a		100	983.7±116.99a	681.3±62.11a
Forest	1	/	/	Grassland	1	/	/
	5	/	/		5	/	/
	10	/	/		10	/	/
	20	54±13.46c	46.2±8.01c		20	35.25±3.29c	25±3.33b
	50	327±45.38b	213.22±35.19b		50	204.25±25.04b	90.5±16.7a
	100	790.11±143.46a	408.7±64.55a		100	327.8±46.15a	223.13±35.43a
Road margins	1	/	/				
	5	/	/				
	10	60.13±10.54c	42±5.22c				
	20	152.78±24.49c	81.8±10.69c				
	50	1056.6±68.66b	672.4±78.04b				
	100	2940.1±265.35a	1820.7±137.18a				

/: the A. artemisiifolia plants in the treatment died and did not produce seeds. Different letters indicate significant differences between different seeding number under the same seeding method (P< 0.05). In all habitats, the results varied significantly among seeding methods (P< 0.05).

**Table 3:** Number of newly established *Ambrosia artemisiifolia* in different habitats in the second year (plants m^-2^, Mean ± SE).

Habitats	Seeding number	Seeding method		Habitats	Seeding number	Seeding method	
		Aggregation	Dispersion			Aggregation	Dispersion
River banks	1	/	/	Farmland	1	/	/
	5	/	/		5	/	/
	10	15.76±2.16a	11.28±1.67a		10	6.11±1.24a	8.17±0.96a
	20	18.69±3.52a	22.12±4.14a		20	20.8±2.98a	18.84±2.15a
	50	38.46±4.11a	25.44±3.12b		50	55.34±8.77a	48.79±10.12a
	100	50.6±6.32a	37.78±5.21b		100	61.78±6.68a	53.21±4.5a
Forest	1	/	/	Grassland	1	/	/
	5	/	/		5	/	/
	10	/	/		10	/	/
	20	11.5±1.16a	11.78±0.84a		20	13.5±1.08a	9.59±0.83a
	50	25.75±3.95a	22.44±2.46a		50	16.18±2.1a	19.75±2.49a
	100	45.12±5.8a	43.78±3.88a		100	23.86±3.12a	18.89±2.61a
Road margins	1	/	/				
	5	/	/				
	10	17.58±2.3a	14.16±2.18a				
	20	30.26±1.97a	22.45±3.01a				
	50	61.71±3.45a	35.41±6.63b				
	100	77.14±8.36a	58.75±8.21b				

/: No A. artemisiifolia established. Different letters indicate significant differences between different seeding method under the same seeding number (P< 0.05).

**Figure 3:**
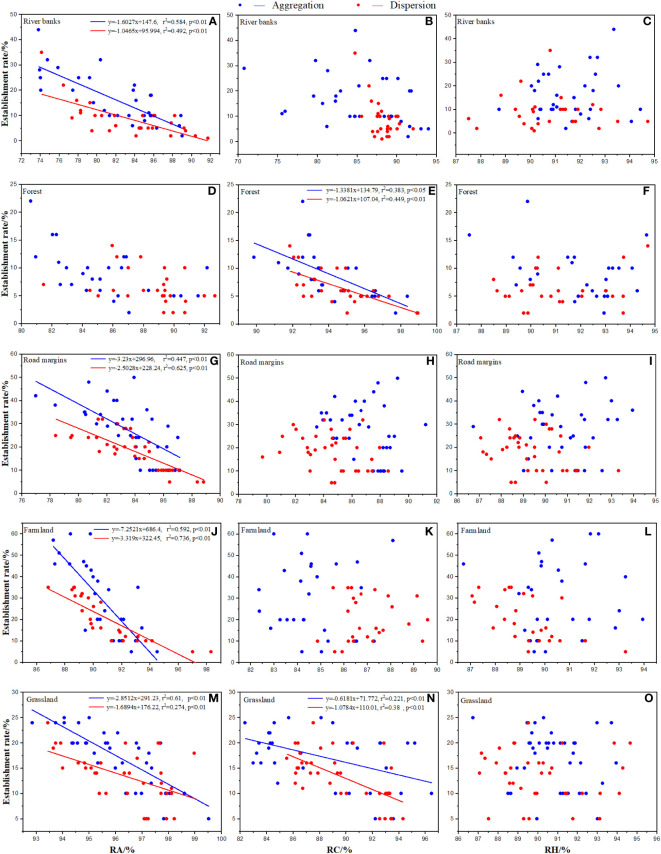
Effects of native species in each habitat on establishment rate of *Ambrosia artemisiifolia*. Trendline fitting indicates a significant effect, while trendline not fitting indicates no significant effect. RA: Total relative abundance of native species. RC: Total relative coverage of native species. RH: Total relative plant height of native species. **(A, D, G, J, M)** Effects of RA in each habitat on the establishment rate of *Ambrosia artemisiifolia*. **(B, E, H, K, N)** Effects of RC in each habitat on the establishment rate of *Ambrosia artemisiifolia*. **(C, F, I, L, O)** Effects of RH in each habitat on the establishment rate of *Ambrosia artemisiifolia. misiifolia*.

**Figure 4:**
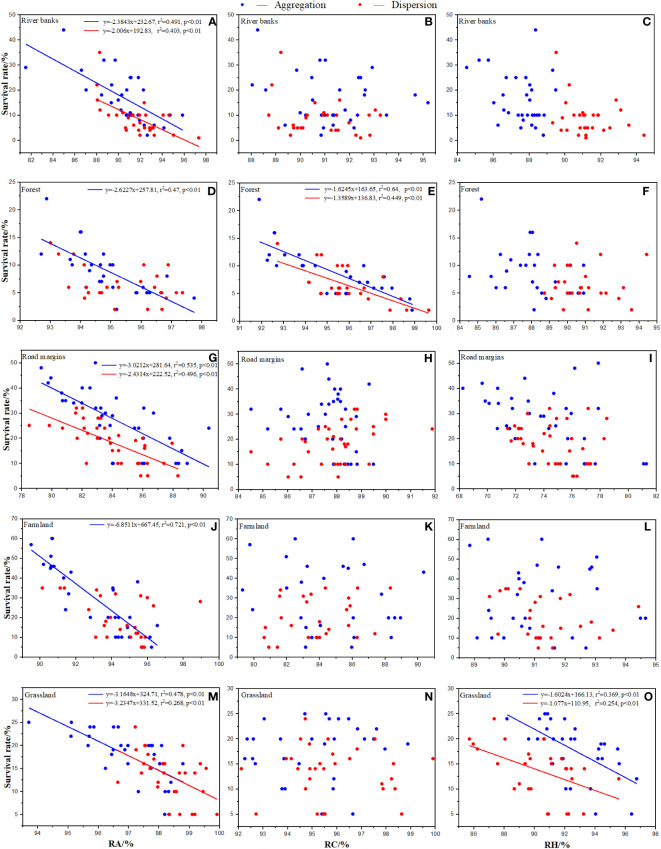
Effects of native species in each habitat on survival rate of *Ambrosia artemisiifolia*. Trendline fitting indicates a significant effect, while trendline not fitting indicates no significant effect. RA: Total relative abundance of native species. RC: Total relative coverage of native species. RH: Total relative plant height of native species. **(A, D, G, J, M)** Effects of RA in each habitat on the survival rate of *Ambrosia artemisiifolia*. **(B, E, H, K, N)** Effects of RC in each habitat on the survival rate of *Ambrosia artemisiifolia*. **(C, F, I, L, O)** Effects of RH in each habitat on the survival rate of *Ambrosia artemisiifolia*.

In random effects, relative abundance (RA) in each habitat, relative coverage (RC) and its interaction with RA in forests, and relative height (RH) and its interaction and RA in grassland had significant negative effects on establishment and survival rates (**Figures 3**, **4**). None of the random effects significantly affected the total number of seeds in a population.

### Effects of propagule pressure on the establishment and survival

At the seedling stage (SS), there were significant differences in the establishment rate of *A. artemisiifolia* among the treatments with different seeding numbers (**Figures 2A-E**). No plants germinated in the wasteland, established with 1 or 5 seed treatments in any habitat, or were successfully established in 10 seed treatment in forest. With an increase in the number of propagules, the establishment rate significantly increased, and the establishment rate of farmland and road margins was very high, up to 44%. Among the different sowing methods, the establishment rate of aggregated sowing was higher than that of dispersed sowing, and the difference was significantfor the 50 and 100 seed treatments.

At the mature stage (MS), there were significant differences in plant survival rates among treatments with different sowing amounts in different habitats (**Figures 2F-J**). The survival rate of plants in the road margins was the highest (27.5%) while the survival rate of plants in the grassland was the lowest. No plants survived in 10 seed treatment. Among the different seeding methods, only the 50 and 100 seed treatments on river banks and road margins, respectively, were significantly different.

### Seed number and plant number dynamics between current and following years

At the MS, all the surviving *A. artemisiifolia* were able to successfully produce seeds. By comparing the total number of seeds in each habitat, with an increase in the number of sows, the total number of seeds in the population significantly increased with increasing seeding. The number of seeds from the aggregated sowing method was significantly higher than from the dispersed sowing method (**Table 2**).

At the SS of the second year, the number of newly established *A. artemisiifolia* plants increased in all treatments with increasing seeding. On river banks and road margins, the number of newly established plants with aggregated sowing in the 50 and 100 seed treatments were significantly higher than that with dispersed sowing (**Table 3**).

### Effects of native species on the establishment and survival

At the SS, the effects of the native species in each habitat were significantly different. Except for the forest, RA in the other habitats had a significant negative effect on the establishment rate of *A. artemisiifolia* (**Figures 3A, D, G, J, M**). RC had a significant negative effect on the establishment rate of *A. artemisiifolia* in the forest and grassland habitats (**Figures 3B, E, H, K, N**). For example, in forest, the RC of *Phlomis umbrosa* and *Arctium lappa* reached 29.03 ± 2.35% and 25.81 ± 3.76%, respectively, 4–5 times that of *A. artemisiifolia* (**Supplementary Table A.1**). The RH had no significant effect on the establishment rate of *A. artemisiifolia* (**Figures 3C, F, I, L, O**).

At the MS, the effects of the native species in each habitat were significantly different. RA had a significant negative effect on the survival rate of *A. artemisiifolia* except for the dispersed sowing treatment in the forest and farmland (**Figures 4A, D, G, J, M**). RC had a significant negative effect on the establishment rate of *A. artemisiifolia* only in forest (**Figures 4B, E, H, K, N**), while the RC of *Phlomis umbrosa* and *Arctium lappa* reached 29.15 ± 2.3% and 23.35 ± 1.65%, respectively, 3–4 times that of *A. artemisiifolia*. RH had a significant negative effect on the survival rate of *A. artemisiifolia* only in the grasslands (**Figures 4C, F, I, L, O**). For example, the RH of *Chenopodium album*, *Artemisia annua*, and *Cannabis sativa* was 11.79 ± 2.7%, 10.8 ± 1.45%, and 10.09 ± 2.73%, respectively, 1.5 times that of *A. artemisiifolia* (**Supplementary Table A.1**).

### Minimum number of seeds required for successful establishment and minimum number of plants required for the population growth

**Figure 5:**
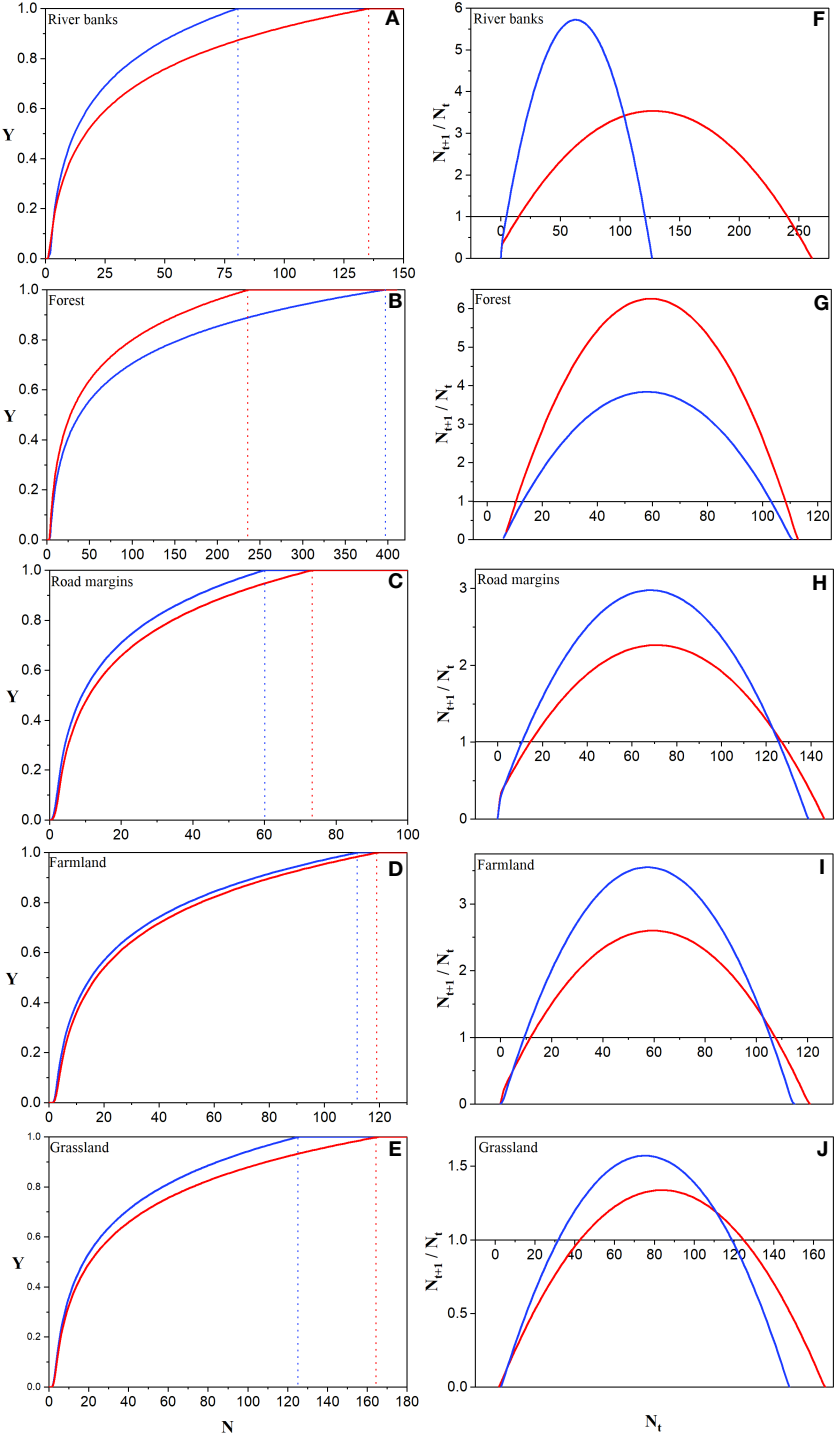
Prediction of MNS **(A-E)** and MNP **(F-J)** in different habitats under different treatments. The solid blue line represents aggregated sowing, and the dashed blue line corresponds to the *N* value when the establishment rate (*Y*) is 1. The solid red line represents the dispersed sowing, and the dotted red line corresponds to the *N* value when the establishment success rate (*Y*) is 1.

The MNS of *A. artemisiifolia* varied significantly among habitats (**Figures 5A-E**). The MNS required by river banks were 81 (aggregation) and 163 (dispersion), forest were 398 (aggregation) and 238 (dispersion), road margins were 60 (aggregation) and 74 (dispersion), farmland were 113 (aggregation) and 120 (dispersion), and grassland were 126 (aggregation) and 166 (dispersion).

The MNP of *A. artemisiifolia* varied significantly among the habitats (**Figures 5F-J**). The MNP required by river banks were 5 (aggregation) and 16 (dispersion), forest were 14 (aggregation) and 11 (dispersion), road margins were 11 (aggregation) and 15 (dispersion), farmland were 10 (aggregation) and 12 (dispersion), and grassland were 32 (aggregation) and 43 (dispersion).

### Water threshold required for establishment and survival

**Figure 6:**
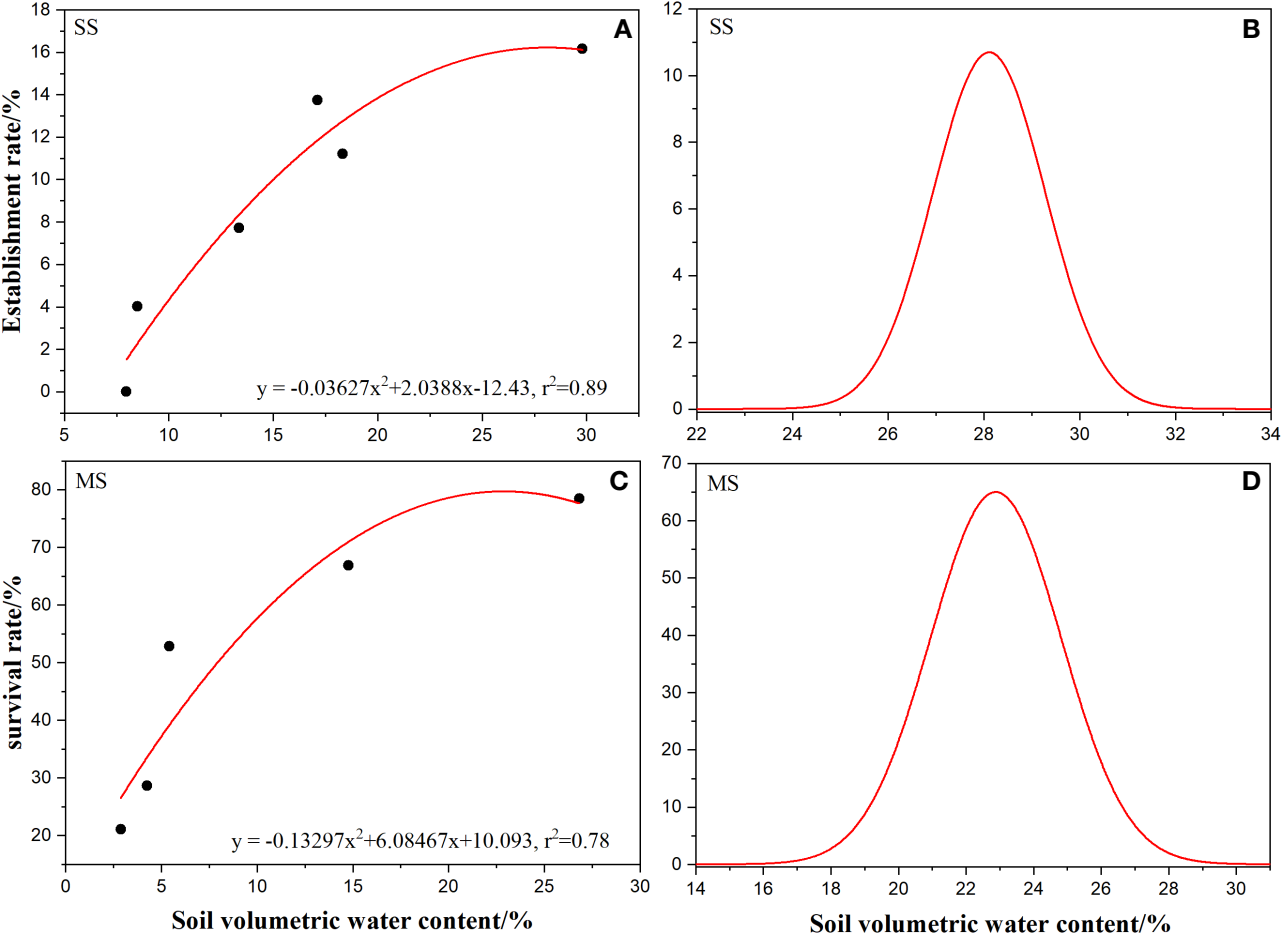
Relationship between establishment rate and survival rate of *Ambrosia artemisiifolia* and soil volumetric water content and Gaussian fitting curve. **(A)** Regression of establishment rate and soil volume moisture content at seedling stage. **(B)** Gaussian curves of fitted establishment rate and soil volumetric water content. **(C)** Regression of survival rate and soil volume moisture content at seedling stage. **(D)** Gaussian curves of fitted survival rate and soil volumetric water content. The black dots are showing in A and C represents the actual value.


Regression analysis of the measured data of soil temperature and volumetric water content showed that soil volumetric water content had a significant positive effect on the establishment rate and survival rate of *A. artemisiifolia* (**Figures 6A, C**), whereas soil temperature had no significant effect. According to the conversion relationship between the quadratic equation and the Gaussian model, the Gaussian regression equation (Equation 5) of soil volumetric water content and establishment rate at the SS, and the Gaussian regression equation (Equation 6) of soil volumetric water content and survival rate at the MS were obtained.

(5) $$Y_{e}=1.07\text{ exp }\left[-0.5{\left(x-28.11\right)}^{2}/1.38\right]\;$$

(6) $$Y_{s}=0.65\text{ exp }\left[-0.5{\left(x-22.88\right)}^{2}/3.67\right]$$

According to Equation 5, the optimal soil volumetric water content for the establishment of *A. artemisiifolia* is 28.11%, the tolerance value is 1.38%, the threshold range of soil volumetric water content required for establishment is 25.77–30.45%, and the optimal range is 26.94–29.28% (**Figure 6B**). Conversely, according to Equation 6, the optimal soil volumetric water content for *A. artemisiifolia* survival at MS is 22.88%, the tolerance value is 3.67%, the threshold range of soil volumetric water content required for survival is 15.54–30.22%, and the optimal range is 19.21–26.55% (**Figure 6D**).

## Discussion

### Successful establishment of *A. artemisiifolia* requires numerous seeds

Prediction, rapid and accurate monitoring, and effective prevention and control of the invasion of *A. artemisiifolia* remain challenging. *A. artemisiifolia* can achieve rapid adaptive evolution under future climate change scenarios (Sun and Roderick, 2019) and has spread to Europe, North America, and Asia (Skjøth et al., 2019; Xian et al., 2023). Although studies have found that roadside and river banks are the most vulnerable habitats to invasion by *A. artemisiifolia* (Lemke et al., 2019), the degree of probability of invasion, going from individual establishment to population outbreak after *A. artemisiifolia* spreads to new habitats, remains unclear.

When invasive alien plants spread to new habitats, their propagules either die or become dormant owing to the restrictions of environmental factors. Therefore, numerous single introductions of propagators will increase the survival probability of invasive species and contribute to their successful establishment (Wittmann et al., 2014). *A. artemisiifolia* seeds exhibit a high degree of dormancy; because of secondary dormancy, *A. artemisiifolia* seeds can remain alive in the soil for decades. Seed dormancy can be broken by wet, dark stratification at 4°C for 2 weeks to obtain about 75% of germination (Baskin and Baskin, 1987; Fumanal et al., 2006). Thus, dispersal by *A. artemisiifolia* into new habitats requires many seeds to ensure establishment.

In this study we found that for the same seed treatment, the risk level of successful establishment of *A. artemisiifolia* in each habitat was as follows: road margins> river banks> farmland> grassland> forest. The MNS was lowest at road margins at 60 seeds and highest in forests at 398 seeds. MNS in river banks, farmland, grassland, and other habitats was in the range of 80–166 seeds. The higher the MNS of the habitat, the lower the potential risk of an outbreak upon arrival. Therefore, the successful establishment of *A. artemisiifolia* is the result of the accumulation of more seeds, rather than immediate invasion once it reaches the new habitat.

The distribution of *A. artemisiifolia* in the Yili Valley has an obvious habitat bias. Except for the native areas in this study, the distribution of bias in other habitats was small or difficult to invade (Zhao et al., 2022). Although only six typical habitats of *A. artemisiifolia* distribution were selected as research sites in this study, they were scattered throughout the study area, and the habitats under different spatial distribution comprehensively represented the habitat types of *A. artemisiifolia* growth. Therefore, the monitoring of *A. artemisiifolia* should focus on the above habitats. *A. artemisiifolia* mainly relies on vehicle cargo transport and drifting with currents to achieve long-distance diffusion. Roadsides, river banks, and ditches are accessible habitats after long-distance diffusion (Joly et al., 2011; Pinke et al., 2011). MNS is relatively small (60 seeds for road margins and 81 for river banks), which should be prioritized for monitoring. In other habitats where the MNS is larger (forest, 238; farmland, 113; grassland, 126), the risk of successful outbreaks is lower. If only a small number of seeds are dispersed in such habitats over long distances through excretion by animals after feeding or via pedestrians, animals, and vehicles; monitoring is not vital in this case.

### Once established, *A. artemisiifolia* needs only a few plants to grow its population

The successful reproduction of sexual plants after spreading to new habitats is an important manifestation of adaptation, in which the success of reproduction and mating is crucial. In low-density populations, the Allee effect is caused by inbreeding depression, an inability to attract or find a mate, or an inability to repel predators (Courchamp et al., 2008). There are four stages of the biological invasion process: (1) arrival, (2) establishment, (3) spread, and (4) impact on the environment and economy (Lockwood et al., 2007); Allee effects are important during establishment and spread (Taylor and Hastings, 2005). Especially for wind-pollinated invasive plants, smaller and more isolated populations limit growth and reduce invasive ability due to stronger Allee effects caused by pollen limitation (Liebhold and Tobin, 2008).

*A. artemisiifolia* relies mainly on wind pollination and is monoecious and cross-flowered (Essl et al., 2015). The Allee effect likely occurs in low-density *A. artemisiifolia* populations that spread to new habitats. In this study, at least five plants were required to reproduce successfully on river banks, and the MNP required to reproduce successfully in habitats outside grassland habitat was in the range of 10–16 plants. This suggests that *A. artemisiifolia* has a low demand for the number of plants needed to achieve population growth, and a high risk of outbreak hazards once successfully established.

Pollen production varies among *A. artemisiifolia* plants from 0.1 to 3.8 billion pollen grains per plant (Fumanal et al., 2007; Smith et al., 2013), according to plant size. The pollen viability and stigma receptive period of *A. artemisiifolia* are approximately 20 and 16 days, respectively, and maintain strong receptivity for approximately 7 consecutive days, with pollen viability of up to 92% (Hao et al., 2015). As the flowers of *A. artemisiifolia* are uniovulate, plants at lower densities capture sufficient pollen for a high seed set (Friedman and Barrett, 2008). However, there are records of *A. artemisiifolia* pollen in air samples hundreds of kilometers from the nearest population, indicating the long-distance travel of the *A. artemisiifolia* pollen (Marco et al., 2006; Stępalska et al., 2020). These characteristics give *A. artemisiifolia* a strong probability of successful pollination; therefore, only a small number of plants are required for successful reproduction.

It has been argued that natural selection may favor the evolution of selfing in non-native ranges, as invading species frequently have small initial population sizes (Razanajatovo et al., 2016). In North America, *A. artemisiifolia* is highly self-incompatible and has a high outcrossing rate (Friedman and Barrett, 2008). However, Li et al. (2012) demonstrated no shift from outcrossing to selfing during *A. artemisiifolia* invasion in China. The maintenance of high outcrossing rates in colonizing populations of *A. artemisiifolia* is likely facilitated by the production of wind-borne pollen (Friedman and Barrett, 2011). Many high-quality seeds can be obtained by a breeding mode dominated by outcrossing, which guarantees a high level of genetic diversity in *A. artemisiifolia* to limit genetic bottlenecks or other restrictions in the initial stage of invasion, which is conducive to stable population growth.

### Population aggregation is more conducive to establishment and breeding success of *A. artemisiifolia* than dispersion

The aggregated distribution of plants is associated with alleviating biological and abiotic environmental stresses (such as drought, low temperatures, wind, and animal feeding) and improving their survival and reproductive ability. Examples include density-dependent hybridization (Schmitt et al., 1987), fairy circles (Getzin et al., 2016), intraspecific facilitation (Reijers et al., 2019), and responses to amplifying and alleviating the effects of environmental pressures (Hodgson et al., 2017; Tamura et al., 2020).

The success of Invasive alien plants depends on the degree of seed and plant aggregation (Moore et al., 2003). The aggregation effect of seed germination is the embodiment of the cluster advantage. When more than one species is involved, a lack of seed clustering allows a second species to invade and possibly outcompete the first species at a fine scale (Velázquez et al., 2014). Seeds of the same species sense each other through the release of allelopathic substances (Renne et al., 2014) and gain competitive advantages over the seeds of other species through strategies such as rapid germination (Byun, 2023).

The germination rate of plants with an aggregated distribution was higher (Ghazian et al., 2021). After establishment, the interspecific competitiveness of seedlings is enhanced based on the advantages of aggregation, which can alleviate competition among native species and significantly increase their survival rate (Čuda et al., 2015; El-Barougy et al., 2020). However, for sexual plants, aggregated plant distribution is more conducive to the successful reproduction of pollen adjacent to hetero-plants, thus compensating for the insufficient plant number in the population. Therefore, an increase in the degree of aggregation improves individual fitness and affects population growth (Kanarek et al., 2013).

In this study, under the same seed treatment, the establishment and survival rates of *A. artemisiifolia* plants with an aggregated distribution were higher than those with a dispersed distribution, especially for 50 and 100 seed treatments. *A. artemisiifolia* mainly relies on gravity to achieve close-range spread (Essl et al., 2015). Owing to the large number and high density of aggregated distributions of *A. artemisiifolia* plants, the final seed number per unit area was large, which ensured a stable seed supply and achieved stable population growth in the second year. Although the number of seeds per unit area of dispersed *A. artemisiifolia* was significantly lower than that of the aggregated sowing, most seeds scattered near the plant after maturity and had an aggregation effect, which resulted in higher germination and establishment rates in the second year, thus achieving rapid population growth. Therefore, aggregated seed distribution lays the foundation for the rapid formation of the cluster growth advantage of *A. artemisiifolia*.

### Water is a prerequisite for the establishment of *A. artemisiifolia*

Habitat conditions play a fundamental role by influencing the invasion process and the composition of native species. Water is an important factor affecting species distribution. Indeed, precipitation contributes more than 50% to the potential distribution of *A. artemisiifolia* (Liu et al., 2021). Simulations on the effect of different precipitation levels on the growth of *A. artemisiifolia*, found that *A. artemisiifolia* was highly adaptable to drought (Leiblein and Lösch, 2011; Leskovšek et al., 2012). However, temperature had no significant effect on the growth and distribution of *A. artemisiifolia* in the Yili Valley (Dong et al., 2020).

In the native range of *A. artemisiifolia*, precipitation in the distribution area is approximately 1,000 mm, and 800 mm in invaded ranges such as France and Austria, respectively (Essl et al., 2015). In Xinyuan County, the main distribution area of *A. artemisiifolia* in the Yili Valley, the average annual precipitation is 417.6 mm, with the maximum reaching 500 mm. *A. artemisiifolia* invaded the Yili Valley in 2010. Since 2014, the area occupied by these species has rapidly increased; by 2017, these two species occupied 1,322 km^2^ (Dong et al., 2020).

In this study, soil water volume had a significant positive effect on the establishment rate of *A. artemisiifolia*. Although the average annual precipitation in the study area was lower than that in the native and invasive areas in Europe, the water supply on river banks or ditches was sufficient and stable, and the water supply on roadsides could also meet the water demand for germination, establishment, and survival of *A. artemisiifolia*. The soil volumetric water content was the lowest in the wasteland, where *A. artemisiifolia* failed to germinate. This suggests that water availability is a prerequisite for the establishment of *A. artemisiifolia* seeds after dispersal into new habitats.

### After invasive plant seeds successfully overcome the restrictions imposed by abiotic environmental factors, their establishment and population growth must overcome restrictions imposed by competition with native species

After the seeds of invasive plants reach a new habitat and successfully overcome the restriction of abiotic environmental factors, competition with native species has a gradually significant effect on establishment and population growth (Blackburn et al., 2011). Native community diversity is one of the main factors affecting invasiveness (Elton, 1958). Bioimpedance occurs when native species in a local community occupy the niche of an invasive species, thereby reducing its survival and fitness (Levine et al., 2004). Higher species diversity can significantly reduce the probability of the successful establishment and survival of invasive species (Vilà et al., 2011). *A. artemisiifolia* is gnot a strong competitor in less disturbed habitats, especially in the early invasion stage; its abundance and coverage are not dominant, and it cannot effectively compete with many of the native species it encounters (Bazzaz, 1968; Gentili et al., 2015).

In the present study, the influence of native plants on the establishment and survival of *A. artemisiifolia* was mainly reflected in the abundance of native species and the dominant native species with high plant heights and high biomass. In the forest, there were abundant native species, and the plant coverage of perennial plants such as *Phlomis umbrosa* and *Arctium lappa* was high, especially funder aggregated treatment of *A. artemisiifolia*, where seedlings provide shelter, affecting establishment. This also explains why the MNS of forests was the highest among all habitats, and the MNS and MNP of aggregated distribution was greater than those for dispersed distribution. Grassland was similar to forest in that native species were abundant and due to the height of native *C. album*, *A. annua* and *C. sativa*, although *A. artemisiifolia* could successfully establish, it could not form an effective clustering advantage over the growth period. Therefore, all *A. artemisiifolia* plants treated at a low density in the grassland (10) died at MS. The proportion of viable plants in the high-density treatment (50 and 100) was the lowest in all habitats.

In other habitats, although the abundance of native species had a significant negative effect on the establishment and survival of *A. artemisiifolia*, due to the small number and variety of native species, no species were significantly larger than *A. artemisiifolia* in coverage or height. Owing to the advantages of cluster growth and tall plants, interactions such as interspecies competition could not completely exclude *A. artemisiifolia* from achieving successful establishment and survival.

## Conclusion

This study has shown that the successful invasion of *A. artemisiifolia* is not likely achieved following dispersal by isolated propagules to new habitats, but rather when the seeds are concentrated by aggregation in habitats with sufficient water supply and few native species, such as river banks, ditches, and roadsides. Therefore, monitoring of these high invasion risk areas should be strengthened to prevent invasion. Once established, *A. artemisiifolia* can successfully reproduce and achieve rapid population growth in the second year of invasion. When found, *A. artemisiifolia* plants should be completely removed to avoid the dispersal of seeds to surrounding areas and continued invasion.

## Data availability statement

The raw data supporting the conclusions of this article will be made available by the authors, without undue reservation.

## Author contributions

WZ conceived this study, performed data analyses, and wrote the manuscript. HW and ZH collected data of this study. TL led and coordinated the project. All authors read and approved the final manuscript. All authors contributed to the article and approved the submitted version.
