# Supplementary figures and images for: Factors affecting establishment and population growth of the invasive weed Ambrosia artemisiifolia
Source: Front Plant Sci. 2023 Sep 22;14:1251441. doi: 10.3389/fpls.2023.1251441 (PMC10556694; doi:10.3389/fpls.2023.1251441)

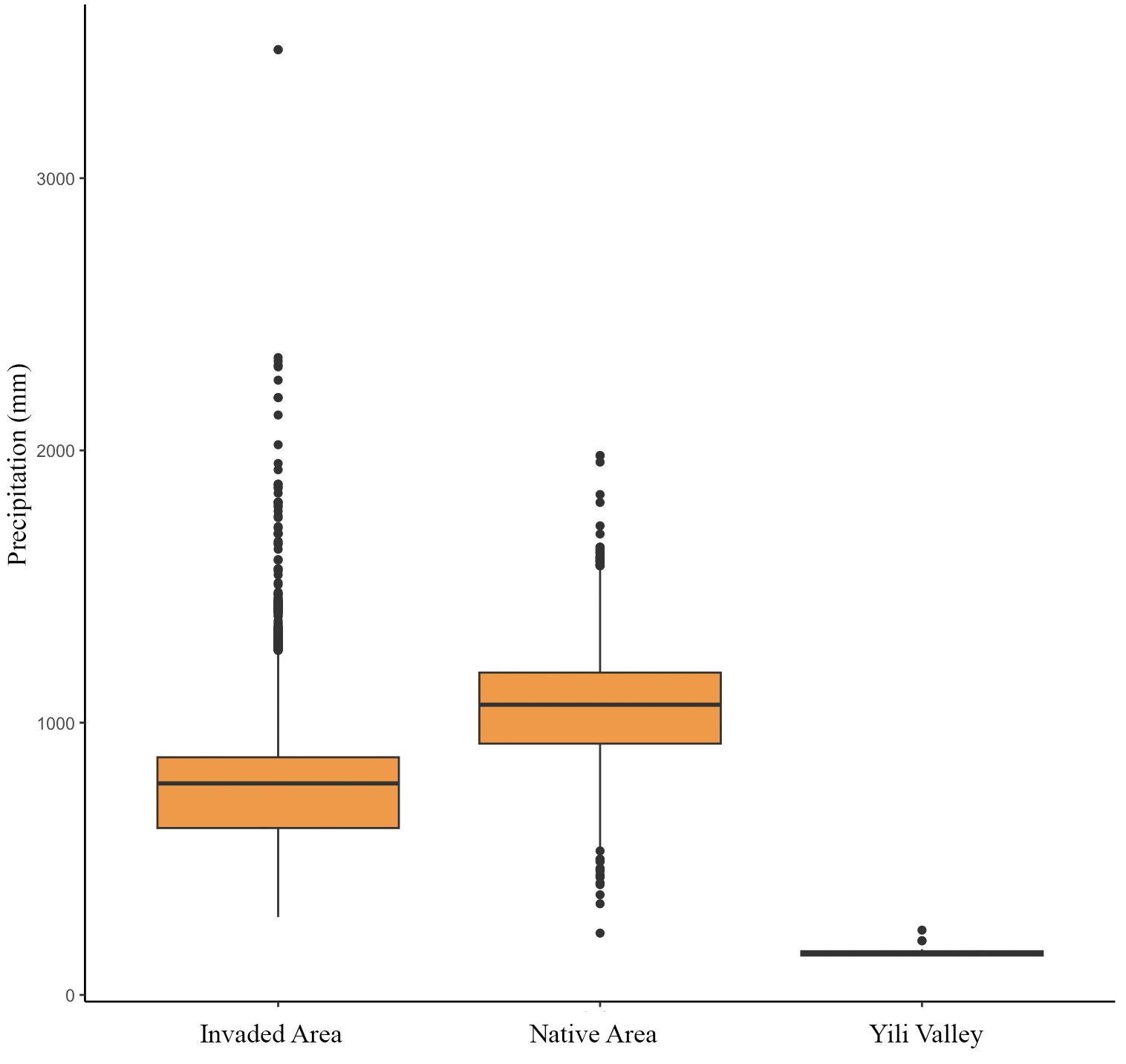

Supplement: Supplementary file 1 [file Image_1.tif]
