# Supplementary material for: Factors affecting establishment and population growth of the invasive weed Ambrosia artemisiifolia
Source: Front Plant Sci. 2023 Sep 22;14:1251441. doi: 10.3389/fpls.2023.1251441 (PMC10556694; doi:10.3389/fpls.2023.1251441)
Supplement: Supplementary file 2 [file Table_1.docx]

**Supplementary** **Table A1.** Composition and distribution characteristics of native species in each habitat

| Stage | Habitats | Species | RF% | RA% | RC% | RH% | Species | RF% | RA% | RC% | RH% |
| --- | --- | --- | --- | --- | --- | --- | --- | --- | --- | --- | --- |
| SS | River banks | *Lolium perenne* | 11.44±1.36 | 18.23±1.98 | 10.17±0.55 | 9.04±0.84 | *Echinochloa crusgali* | 7.05±0.81 | 6.08±0.96 | 10.17±1.19 | 10.84±1.35 |
|  |  | *Festuca ovina* | 11.7±1.11 | 11.6±0.95 | 10.17±2.03 | 9.64±0.66 | *Artemisia leucophylla* | 7.31±1.22 | 6.63±0.74 | 10.17±1.05 | 7.83±0.69 |
|  |  | *Ambrosia artemisiifolia* | 13.3±1.85 | 11.05±2.03 | 10.17±1.19 | 6.02±0.79 | *Plantago asiatica* | 6.91±0.76 | 7.18±1.25 | 8.47±0.71 | 6.63±0.55 |
|  |  | *Geranium wilfordii* | 11.3±2.15 | 13.81±1.64 | 6.78±0.77 | 8.43±0.82 | *Phragmites australis* | 3.59±0.31 | 2.76±0.43 | 6.78±0.85 | 15.06±2.97 |
|  |  | *Elytrigia repens* | 10.24±1.28 | 11.05±1.73 | 8.47±0.59 | 10.24±0.92 | *Polygonum aviculare* | 5.98±0.46 | 4.42±0.38 | 8.47±0.63 | 7.23±0.8 |
|  |  | *Chenopodium album* | 11.17±1.93 | 7.18±1.1 | 10.17±1.05 | 9.04±0.81 |  |  |  |  |  |
|  | Forest | *Phlomis umbrosa* | 13.41±2.89 | 4±0.72 | 29.03±2.35 | 10.53±2.16 | *Ambrosia artemisiifolia* | 15.77±1.1 | 8±0.58 | 6.45±0.85 | 8.42±0.73 |
|  |  | *Galium odoratum* | 15.77±2.81 | 28.8±3.15 | 6.45±0.89 | 5.26±0.61 | *Geranium wilfordii* | 4.73±1.12 | 8.8±0.92 | 8.06±0.75 | 11.58±1.14 |
|  |  | *Arctium lappa* | 13.88±1.79 | 4.8±0.39 | 25.81±3.76 | 10.53±1.57 | *Conyza canadensis* | 6.31±0.79 | 6.4±0.56 | 6.45±0.82 | 13.68±2.19 |
|  |  | *Setaria viridis* | 15.77±1.85 | 20±2.36 | 6.45±0.71 | 10.53±1.84 | *Plantago asiatica* | 5.52±0.25 | 7.2±0.61 | 6.45±0.96 | 12.63±1.57 |
|  |  | *Poa annua* | 8.83±1.15 | 12±1.38 | 4.84±0.98 | 16.84±2.79 |  |  |  |  |  |
|  | Road margins | *Ambrosia artemisiifolia* | 26.18±3.51 | 16.13±2.33 | 13.42±1.88 | 11.59±2.91 | *Geranium wilfordii* | 10.47±1.2 | 12.9±2.58 | 16.11±2.68 | 14.49±3.65 |
|  |  | *Conyza canadensis* | 16.49±2.14 | 17.2±3.11 | 13.42±2.15 | 14.49±2.75 | *Echinochloa crusgali* | 11.52±2.46 | 8.6±1.51 | 20.13±3.42 | 11.59±2.92 |
|  |  | *Elytrigia repens* | 13.09±2.5 | 11.83±2.47 | 16.78±2.35 | 18.84±3.97 | *Setaria viridis* | 13.09±2.5 | 15.05±3.46 | 6.71±1.41 | 11.59±2.18 |
|  |  | *Eleusine indica* | 9.16±0.37 | 18.28±2.79 | 13.42±1.88 | 17.39±3.04 |  |  |  |  |  |
|  | Farmland | *Medicago sativa* | 22.73±4.1 | 46.15±4.62 | 17.65±2.88 | 25±2.7 | *Galium odoratum* | 18.18±2.86 | 11.28±2.05 | 11.76±2.19 | 6±0.53 |
|  |  | *Ambrosia artemisiifolia* | 22.73±3.76 | 10.26±1.43 | 14.71±1.59 | 10±1.3 | *Artemisia annua* | 7.95±0.55 | 5.13±0.51 | 11.76±1.38 | 16±2.8 |
|  |  | *Bromus japonicus* | 11.36±2.64 | 12.82±2.81 | 14.71±2.84 | 15±2.15 | *Eleusine indica* | 7.95±0.93 | 6.67±0.84 | 11.76±2.37 | 13±3.2 |
|  |  | *Chenopodium album* | 9.09±0.91 | 7.69±1.77 | 17.65±3.16 | 15±2.4 |  |  |  |  |  |
|  | Grassland | *Poa annua* | 9.65±1.93 | 12.05±2.89 | 6.94±1.44 | 11.63±1.97 | *Festuca ovina* | 7.7±1.13 | 6.63±0.65 | 6.94±0.44 | 9.88±1.29 |
|  |  | *Artemisia annua* | 8.37±1.36 | 10.24±1.39 | 8.68±0.56 | 9.3±1.56 | *Sonchus oleraceus* | 7.7±0.92 | 9.04±1.14 | 5.21±0.32 | 8.72±1.02 |
|  |  | *Galium odoratum* | 9.62±1.12 | 9.64±1.24 | 10.42±2.67 | 2.91±0.67 | *Ambrosia artemisiifolia* | 9.62±1.16 | 3.61±0.78 | 6.94±0.85 | 5.81±0.53 |
|  |  | *Cannabis sativa* | 6.74±0.77 | 6.63±0.65 | 10.42±1.87 | 8.72±1.02 | *Elymus dahuricus* | 6.74±0.44 | 3.01±0.82 | 5.21±0.84 | 10.47±1.63 |
|  |  | *Chenopodium album* | 6.45±0.82 | 6.02±0.96 | 12.15±1.78 | 7.56±1.25 | *Eleusine indica* | 6.26±1.34 | 5.42±0.76 | 6.94±0.28 | 5.81±0.53 |
|  |  | *Setaria viridis* | 9.62±0.91 | 10.84±1.35 | 5.21±0.83 | 6.4±0.49 | *Cirsium japonicum* | 4.81±0.95 | 7.83±0.84 | 6.94±0.67 | 4.65±0.21 |
|  |  | *Conyza canadensis* | 6.74±0.47 | 9.04±1.45 | 7.99±1.1 | 8.14±0.49 |  | | | | |
| MS | River banks | *Lolium perenne* | 11.19±2.74 | 15.87±2.15 | 10.17±1.96 | 10.45±2.56 | *Plantago asiatica* | 7±0.55 | 12.17±2.13 | 8.47±0.95 | 6.72±1.01 |
|  |  | *Festuca ovina* | 11.86±1.59 | 11.11±1.14 | 10.17±1.59 | 11.57±1.83 | *Elytrigia repens* | 10.38±2.29 | 10.58±1.5 | 8.47±0.98 | 4.85±0.38 |
|  |  | *Ambrosia artemisiifolia* | 13.48±1.16 | 7.94±1.06 | 10.17±1.28 | 9.33±1.16 | *Artemisia leucophylla* | 7.41±0.66 | 7.41±0.72 | 10.17±1.53 | 8.59±0.61 |
|  |  | *Geranium wilfordii* | 10.78±1.84 | 12.7±2.33 | 6.78±0.85 | 8.96±0.71 | *Echinochloa crusgali* | 7.14±0.82 | 5.82±0.69 | 10.17±1.28 | 4.1±0.62 |
|  |  | *Chenopodium album* | 11.32±1.56 | 7.41±0.89 | 10.17±1.15 | 8.96±0.84 | *Polygonum aviculare* | 5.39±0.61 | 5.82±0.47 | 8.47±0.77 | 4.85±0.38 |
|  |  | *Phragmites australis* | 4.04±0.34 | 3.17±0.25 | 6.78±0.51 | 21.64±3.35 |  |  |  |  |  |
|  | Forest | *Phlomis umbrosa* | 13.32±2.88 | 4.31±0.34 | 29.15±2.3 | 8.33±0.33 | *Ambrosia artemisiifolia* | 15.67±1.29 | 5.17±0.42 | 6.45±0.61 | 10±0.82 |
|  |  | *Poa annua* | 9.87±1.46 | 17.24±3.79 | 4.83±0.87 | 22.5±2.15 | *Geranium wilfordii* | 4.7±0.22 | 11.21±1.69 | 8.06±0.54 | 12.5±1.38 |
|  |  | *Arctium lappa* | 12.54±1.85 | 5.17±0.38 | 23.35±1.65 | 8.33±0.57 | *Conyza canadensis* | 6.27±0.52 | 9.48±0.86 | 6.45±0.53 | 12.5±1.09 |
|  |  | *Galium odoratum* | 15.67±3.98 | 21.55±1.72 | 6.45±0.52 | 5±0.37 | *Plantago asiatica* | 6.27±0.49 | 8.62±0.44 | 6.45±0.61 | 11.67±1.23 |
|  |  | *Setaria viridis* | 15.67±2.81 | 17.24±2.37 | 6.45±0.61 | 9.17±0.67 |  |  |  |  |  |
|  | Road margins | *Ambrosia artemisiifolia* | 26.04±2.67 | 14.29±3.41 | 13.42±2.28 | 24.67±3.24 | *Eleusine indica* | 10.42±2.51 | 16.48±3.65 | 13.42±2.28 | 11.04±2.69 |
|  |  | *Conyza canadensis* | 15.63±2.53 | 16.48±3.65 | 13.42±2.76 | 16.88±3.69 | *Echinochloa crusgali* | 11.46±2.33 | 10.99±1.11 | 20.13±2.43 | 6.49±1.56 |
|  |  | *Geranium wilfordii* | 10.42±1.76 | 14.29±2.56 | 16.11±3.7 | 19.48±4.95 | *Setaria viridis* | 13.02±3.8 | 16.48±1.95 | 6.7±1.14 | 5.19±0.82 |
|  |  | *Elytrigia repens* | 13.02±3.8 | 10.99±1.08 | 16.78±2.85 | 16.23±2.66 |  |  |  |  |  |
|  | Farmland | *Medicago sativa* | 22.47±2.91 | 43.2±6.63 | 17.65±2.47 | 21.57±5.27 | *Chenopodium album* | 8.99±1.64 | 7.1±1.79 | 17.65±3.89 | 13.73±3.11 |
|  |  | *Bromus japonicus* | 11.24±1.55 | 20.12±2.34 | 14.71±2.59 | 16.86±4.51 | *Galium odoratum* | 17.98±2.81 | 10.65±1.76 | 11.76±2.34 | 2.35±0.12 |
|  |  | *Ambrosia artemisiifolia* | 22.47±2.88 | 7.69±1.47 | 14.71±1.32 | 10.98±2.39 | *Eleusine indica* | 8.99±1.01 | 5.33±0.83 | 11.76±1.54 | 12.55±1.96 |
|  |  | *Artemisia annua* | 7.87±1.68 | 5.92±1.59 | 11.77±2.46 | 21.96±3.48 |  |  |  |  |  |
|  | Grassland | *Poa annua* | 9.86±0.32 | 15.38±4.51 | 6.94±1.88 | 9.52±1.7 | *Setaria viridis* | 9.86±1.93 | 12.43±2.55 | 5.21±0.84 | 2.13±0.81 |
|  |  | *Chenopodium album* | 6.61±1.59 | 5.33±0.83 | 12.15±2.78 | 11.79±2.7 | *Sonchus oleraceus* | 5.92±0.58 | 9.47±1.16 | 5.21±0.76 | 8.38±1.01 |
|  |  | *Artemisia annua* | 8.58±1.78 | 7.69±0.77 | 8.68±0.65 | 10.8±1.45 | *Eleusine indica* | 6.41±0.97 | 4.14±0.42 | 6.94±0.85 | 9.94±1.18 |
|  |  | *Conyza canadensis* | 6.9±1.35 | 9.47±1.56 | 7.99±0.61 | 9.66±1.09 | *Ambrosia artemisiifolia* | 9.86±1.93 | 2.37±0.39 | 6.94±0.24 | 7.53±0.91 |
|  |  | *Festuca ovina* | 6.41±0.85 | 7.69±1.77 | 10.42±2.58 | 6.39±1.46 | *Elymus dahuricus* | 6.9±0.55 | 4.73±0.87 | 5.21±0.78 | 7.39±0.63 |
|  |  | *Cannabis sativa* | 7.89±1.02 | 5.92±0.98 | 6.94±0.44 | 10.09±2.73 | *Cirsium japonicum* | 4.93±0.65 | 6.51±0.75 | 6.94±0.88 | 5.4±0.73 |
|  |  | *Galium odoratum* | 9.86±0.92 | 8.88±0.67 | 10.42±1.37 | 0.99±0.02 |  | | | | |

**Note:** RF: Total relative frequency of native species. RA: Total relative abundance of native species. RC: Total relative coverage of native species. RH: Total relative plant height of native species. SS: seedling stage. MS: Maturity stage.
