# Supplementary material for: Factors affecting establishment and population growth of the invasive weed Ambrosia artemisiifolia
Source: Front Plant Sci. 2023 Sep 22;14:1251441. doi: 10.3389/fpls.2023.1251441 (PMC10556694; doi:10.3389/fpls.2023.1251441)
Supplement: Supplementary file 3 [file Table_2.docx]

**Supplementary** **Table A2.** The difference and variation degree of soil temperature and volume water content between SS and MS in different habitats.

| Stage | Habitats | Soil temperature (℃) | | Soil volume water content (%) | |
| --- | --- | --- | --- | --- | --- |
|  |  | Mean ± SD | CV | Mean ± SD | CV |
| SS | River banks | 14.59±0.16b | 0.308 | 13.37±0.16d | 0.148 |
|  | Forest | 8.68±0.25d | 0.441 | 13.80±0.16d | 0.174 |
|  | Road margins | 12.92±0.23c | 0.274 | 30.23±0.27a | 0.138 |
|  | farmland | 14.52±0.26b | 0.272 | 14.90±0.35c | 0.362 |
|  | grassland | 17.52±0.40a | 0.350 | 18.32±0.41b | 0.348 |
|  | Wasteland | 16.96±0.32a | 0.289 | 7.97±0.15e | 0.290 |
| MS | River banks | 20.66±0.12c | 0.139 | 14.79±0.09b | 0.335 |
|  | Forest | 24.68±0.16a | 0.174 | 5.19±0.07c | 0.357 |
|  | Road margins | 21.28±0.09b | 0.109 | 27.84±0.15a | 0.139 |
|  | farmland | 21.27±0.08b | 0.101 | 6.02±0.18c | 0.787 |
|  | grassland | 16.13±0.07d | 0.112 | 4.10±0.09c | 0.587 |

**Note:** Since there was no establishment of *Ambrosia artemisiifolia* in the Wasteland, the soil temperature and volumetric water content were not observed during the MS. Different letters indicate significant differences between habitats. SS: seedling stage. MS: Maturity stage.
